# Tetra­pyridinium μ-oxido-di-μ-sulfato-bis­[chloridodioxidomolybdate(VI)]

**DOI:** 10.1107/S1600536810028254

**Published:** 2010-07-24

**Authors:** José A. Fernandes, Ana C. Gomes, Sónia Figueiredo, Sandra Gago, André D. Lopes, Martyn Pillinger, Paulo J. A. Ribeiro-Claro, Isabel S. Gonçalves, Filipe A. Almeida Paz

**Affiliations:** a Department of Chemistry, University of Aveiro, CICECO, 3810-193 Aveiro, Portugal; b Department of Chemistry, University of Algarve, Campus de Gambelas, 8005-139 Faro, Portugal

## Abstract

The title salt, (C_5_H_6_N)_4_[Mo_2_Cl_2_O_5_(SO_4_)_2_], comprises four pyridinium cations for each [(MoClO_2_)_2_(μ-O)(μ-SO_4_)_2_]^4−^ anionic unit. The asymmetric unit consists of three aggregates of the empirical formula. The tetra­anionic bimetallic molybdenum(VI) cluster is unprecedented and contains two sulfate and one oxide bridges. This structure constitutes the first example of a non-polymeric compound with terminal oxide, sulfate and halide ligands bonded to the same metal. The hydrogen bonds connecting the pyridinium cations to the molybdenum clusters are diverse, varying from strong and directional interactions to bifurcated bonds with a subsequent loss of directionality.

## Related literature

For previous studies on dioxidomolybdenum complexes, see: Monteiro *et al.* (2010 ▶); Gago *et al.* (2009 ▶); Pereira *et al.* (2007 ▶); Cunha-Silva *et al.* (2007 ▶). For a description of the Cambridge Structural Database, see: Allen (2002 ▶). For a related tetra­nuclear cluster, see: Clegg *et al.* (1990 ▶). For related sulfato-bridged bimetallic compounds, see: Zhao *et al.* (2006 ▶); Zhang *et al.* (2005 ▶); Wieghardt *et al.* (1989 ▶).
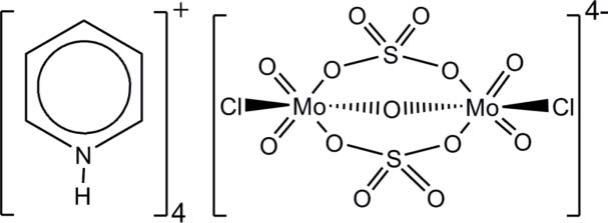


## Experimental

### 

#### Crystal data

(C_5_H_6_N)_4_[Mo_2_Cl_2_O_5_(SO_4_)_2_]
*M* *_r_* = 855.33
Monoclinic, 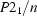

*a* = 10.517 (4) Å
*b* = 49.281 (15) Å
*c* = 17.557 (6) Å
β = 95.07 (3)°
*V* = 9064 (5) Å^3^
*Z* = 12
Mo *K*α radiation
μ = 1.21 mm^−1^
*T* = 150 K
0.03 × 0.02 × 0.01 mm

#### Data collection

Bruker X8 Kappa CCD APEXII diffractometer
Absorption correction: multi-scan (*SADABS*; Sheldrick, 1998 ▶) *T*_min_ = 0.965, *T*_max_ = 0.988
49290 measured reflections
16283 independent reflections
6037 reflections with *I* > 2σ(*I*)
*R*_int_ = 0.255

#### Refinement

*R*[*F* ^2^ > 2σ(*F* ^2^)] = 0.098
*wR*(*F* ^2^) = 0.286
*S* = 0.94
16283 reflections
802 parameters
H-atom parameters constrained
Δρ_max_ = 2.08 e Å^−3^
Δρ_min_ = −1.28 e Å^−3^

### 

Data collection: *APEX2* (Bruker, 2006 ▶); cell refinement: *SAINT-Plus* (Bruker, 2005 ▶); data reduction: *SAINT-Plus*; program(s) used to solve structure: *SHELXTL* (Sheldrick, 2008 ▶); program(s) used to refine structure: *SHELXTL*; molecular graphics: *DIAMOND* (Brandenburg, 2009 ▶); software used to prepare material for publication: *SHELXTL*.

## Supplementary Material

Crystal structure: contains datablocks global, I. DOI: 10.1107/S1600536810028254/tk2692sup1.cif

Structure factors: contains datablocks I. DOI: 10.1107/S1600536810028254/tk2692Isup2.hkl

Additional supplementary materials:  crystallographic information; 3D view; checkCIF report

## Figures and Tables

**Table 1:** Hydrogen-bond geometry (Å, °)

*D*—H⋯*A*	*D*—H	H⋯*A*	*D*⋯*A*	*D*—H⋯*A*
N1_4—H1_4⋯O8_2	0.88	2.18	3.01 (3)	157
N1_5—H1_5⋯O9_1	0.88	2.43	3.15 (2)	139
N1_6—H1_6⋯O12_2	0.88	2.50	3.32 (3)	157
N1_7—H1_7⋯O6_2^i^	0.88	2.11	2.99 (2)	176
N1_8—H1_8⋯O6_3	0.88	2.13	2.90 (2)	146
N1_8—H1_8⋯O10_3	0.88	2.39	3.09 (2)	137
N1_9—H1_9⋯O7_2	0.88	2.03	2.86 (3)	157
N1_9—H1_9⋯O9_2	0.88	2.41	2.94 (2)	119
N1_10—H1_10⋯O4_3	0.88	2.34	3.01 (3)	133
N1_11—H1_11⋯O13_1	0.88	2.04	2.84 (2)	149
N1_11—H1_11⋯O11_1	0.88	2.64	3.18 (2)	121
N1_12—H1_12⋯O5_3	0.88	2.00	2.74 (3)	141
N1_12—H1_12⋯O8_3	0.88	2.45	3.07 (3)	128
N1_13—H1_13⋯O13_1	0.88	1.85	2.722 (18)	173
N1_14—H1_14⋯O8_3	0.88	2.25	3.09 (3)	160
N1_15—H1_15⋯O9_2^ii^	0.88	2.25	3.02 (2)	146

Symmetry codes: (i) 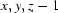; (ii) 
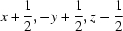
.

**Table 2:** Geometrical parameters (Å,°) for the three crystallographically independent molybdenum clusters

Mo—O1	1.857 (13)–1.969 (14)
Mo—O_terminal_	1.678 (11)–1.717 (12)
Mo—O_sulfato_	2.164 (16)–2.271 (14)
Mo—Cl	2.271 (14)–2.442 (5)
O_terminal_—Mo—O_terminal_	102.2 (7)–104.3 (8)
*cis*-O_terminal_—Mo—O_sulfato_	86.4 (6)–92.6 (7)
*trans*-O_terminal_—Mo—O_sulfato_	162.5 (6)–169.4 (4)
O_terminal_—Mo—O1	96.4 (7)–101.6 (5)
O_terminal_—Mo—Cl	91.2 (5)–97.4 (6)
O_sulfato_—Mo—O_sulfato_	74.8 (5)–79.8 (6)
O_sulfato_—Mo—O1	82 (5)–85.9 (5)
O_sulfato_—Mo—Cl	77.8 (3)–82.6 (3)
O1—Mo—Cl	158 (4)–159.4 (3)
Mo—O1—Mo	149.3 (7)–155 (6)

Notes: O1 stands for the μ-O oxygen atom; O_terminal_ corresponds to O2, O3, O4 and O5; O_sulfato_ corresponds to O6, O7, O10 and O11.

**Fig. 1.**
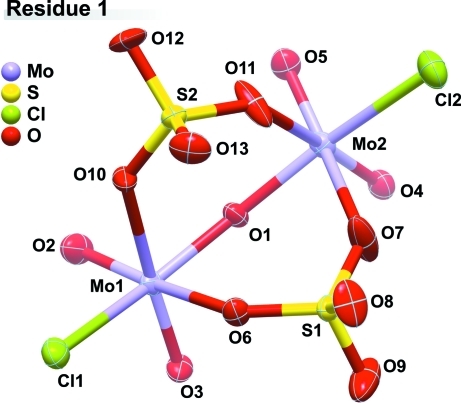
Molecular structure of [(MoClO2)2(µ-O)(µ-SO4)2]4- (residue 1). Thermal ellipsoids are drawn at the 30% probability level. For the ranges of bond lengths (in Å) and angles (in °) of the three crystallographically independent anionic residues see Table 1.

**Fig. 2.**
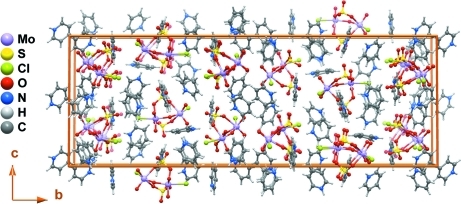
Crystal packing of the title compound viewed in perspective along the [001] direction of the unit cell.

## supplementary crystallographic information

### Comment

In the last few years our combined research groups have been interested in the design, synthesis and detailed structural elucidation of novel catalysts based on dioxomolybdenum complexes (Monteiro *et al.*, 2010; Gago *et al.*, 2009; Pereira *et al.*, 2007; Cunha-Silva *et al.*, 2007). Knowing that compounds of the type [(MoClO_2_*L*_2_)_2_(µ-O)] are important in catalytic olefin epoxidation (Pereira *et al.*, 2007), we were interested in preparing the particular compound with *L* = py (py = pyridine). However, the synthesis of this compound was not feasible. During our most recent efforts to coordinate pyridine to the molybdenum centre, we have isolated the title compound as a secondary product, (C_5_NH_6_)_4_[(MoClO_2_)_2_(µ-O)(µ-SO_4_)_2_], whose structure we would like to report here. Surveying the Cambridge Structural Database (Allen, 2002), only polymeric compounds were found with terminal oxo, sulfato and halo ligands bonded to the same metal. A similar survey for bridging oxo, sulfato and halo ligands yielded only two non-polymeric structures comprising the same tetranuclear cluster of chromium (Clegg *et al.*, 1990). Only three bimetallic compounds with the *M*_2_(µ-O)(µ-SO_4_)_2_ moiety exist (with Ti: Zhang *et al.*, 2005; with Fe: Wieghardt *et al.*, 1989; Zhao *et al.*, 2006).

The asymmetric unit of the title compound comprises three [(MoClO_2_)_2_(µ-O)(µ-SO_4_)_2_]^4-^ anionic complexes which crystallize with twelve charge-balancing pyridinium cations (PyH^+^). Each metallic cluster is composed of two molybdenum(VI) centres, bridged by two sulfato and one oxo ligands. Additionally, each metal centre has two terminal oxo and a chlorido ligand. The coordination geometries of the metallic centres resemble highly distorted octahedra: see Table 1 for the ranges of Mo—O_bridge_, Mo—O_terminal_, Mo—O_sulfato_, and Mo—Cl bond distances. The *cis* and *trans* octahedral angles are deviated from the ideal values [found in the 74.8 (5)–104.3 (7)° and 158.0 (4)–169.4 (6)° ranges, respectively, Table 1]. The "kink" Mo—O—Mo angles range from 149.3 (7) to 155 (6)°.

The overall crystal structure is sustained by the existence of two sub-sets of N^+^—H···O^-^ hydrogen bonding interactions (Fig. 2). On the one hand, several PyH^+^ cations are engaged in strong and rather directional interactions, with the N···O distances ranging from 2.722 (18) to 3.32 (2) Å, and the corresponding <(N—H···O) angles being found between 133 and 176° (Table 2). On the other, four cations are instead interacting with the anionic complexes *via* bifurcated hydrogen bonds which lead to a loss of directionality of the interactions [<(N—H···O) angles in the range of 119–157°], despite the N···O distances being somewhat in a similar range [2.74 (3)–3.18 (2) Å, Table 2].

### Experimental

All chemicals were purchased from commercial sources and used as received without further purification. A solution of pyridine (1.34 ml, 16.6 mmol) in CH_2_Cl_2_ (60 ml) was slowly added drop wise to an aqueous solution (30 ml) of HCl (3.3 mol dm^-3^) containing Na_2_MoO_4_^.^2H_2_O (2.0 g, 8.3 mmol). The biphasic mixture was vigorously stirred for 3 h at ambient temperature. The aqueous phase was separated and concentrated yielding a solid which was dissolved in acetonitrile. The acetonitrile solution was then dried over anhydrous MgSO_4_ (in excess) and evaporated to give a pale-yellow solid. Recrystallization by slow diffusion of diethyl ether into a concentrated acetonitrile solution afforded a yellow crystalline product in 12% yield. Selected FT–IR (ATR, cm^-1^): 941 (vs, Mo═O), 925 (vs, Mo═O), 749 (s, Mo—O—Mo).

### Refinement

Hydrogen atoms bound to carbon and nitrogen were located at their idealized positions and were included in the final structural model in riding-motion approximation with C—H = 0.95 Å and N—H = 0.88 Å. The isotropic thermal displacement parameters for these atoms were fixed at 1.2 times *U*_eq_ of the respective carbon atom.

The 12 crystallographically independent pyridinium cations were found to be severely affected by thermal disorder and were modeled with all nitrogen and carbon atoms having independent isotropic displacement parameters.

The maximum and minimum residual electron density peaks of 2.08 and -1.28 eÅ^-3^, respectively, were located 1.06 Å and 0.78 Å from the Mo1 and Mo2 atoms, respectively, belonging to residue 3.

### Crystal data

**Table:**

(C_5_H_6_N)_4_[Mo_2_Cl_2_O_5_(SO_4_)_2_]	*F*(000) = 5112
*M**_r_* = 855.33	*D*_x_ = 1.880 Mg m^−^^3^
Monoclinic, *P*2_1_/*n*	Mo *K*α radiation, λ = 0.71073 Å
Hall symbol: -P 2yn	Cell parameters from 1387 reflections
*a* = 10.517 (4) Å	θ = 3.9–17.8°
*b* = 49.281 (15) Å	µ = 1.21 mm^−^^1^
*c* = 17.557 (6) Å	*T* = 150 K
β = 95.07 (3)°	Plate, pale-yellow
*V* = 9064 (5) Å^3^	0.03 × 0.02 × 0.01 mm
*Z* = 12	

### Data collection

**Table:**

Bruker X8 Kappa CCD APEXII diffractometer	16283 independent reflections
Radiation source: fine-focus sealed tube	6037 reflections with *I* > 2σ(*I*)
graphite	*R*_int_ = 0.255
ω/φ scans	θ_max_ = 25.4°, θ_min_ = 3.7°
Absorption correction: multi-scan (*SADABS*; Sheldrick, 1998)	*h* = −12→12
*T*_min_ = 0.965, *T*_max_ = 0.988	*k* = −59→59
49290 measured reflections	*l* = −21→21

### Refinement

**Table:**

Refinement on *F*^2^	Primary atom site location: structure-invariant direct methods
Least-squares matrix: full	Secondary atom site location: difference Fourier map
*R*[*F*^2^ > 2σ(*F*^2^)] = 0.098	Hydrogen site location: inferred from neighbouring sites
*wR*(*F*^2^) = 0.286	H-atom parameters constrained
*S* = 0.94	*w* = 1/[σ^2^(*F*_o_^2^) + (0.1162*P*)^2^] where *P* = (*F*_o_^2^ + 2*F*_c_^2^)/3
16283 reflections	(Δ/σ)_max_ = 0.001
802 parameters	Δρ_max_ = 2.08 e Å^−^^3^
0 restraints	Δρ_min_ = −1.28 e Å^−^^3^

### Special details

**Table:**

Geometry. All e.s.d.'s (except the e.s.d. in the dihedral angle between two l.s. planes) are estimated using the full covariance matrix. The cell e.s.d.'s are taken into account individually in the estimation of e.s.d.'s in distances, angles and torsion angles; correlations between e.s.d.'s in cell parameters are only used when they are defined by crystal symmetry. An approximate (isotropic) treatment of cell e.s.d.'s is used for estimating e.s.d.'s involving l.s. planes.
Refinement. Refinement of *F*^2^ against ALL reflections. The weighted *R*-factor *wR* and goodness of fit *S* are based on *F*^2^, conventional *R*-factors *R* are based on *F*, with *F* set to zero for negative *F*^2^. The threshold expression of *F*^2^ > σ(*F*^2^) is used only for calculating *R*-factors(gt) *etc*. and is not relevant to the choice of reflections for refinement. *R*-factors based on *F*^2^ are statistically about twice as large as those based on *F*, and *R*- factors based on ALL data will be even larger.

### Fractional atomic coordinates and isotropic or equivalent isotropic displacement parameters (Å^2^)

**Table:**

	*x*	*y*	*z*	*U*_iso_*/*U*_eq_	
Mo1_1	0.61841 (14)	0.04241 (3)	0.29835 (8)	0.0331 (4)	
Mo2_1	0.86953 (14)	0.08147 (3)	0.21318 (9)	0.0345 (4)	
S1_1	0.7478 (4)	0.10087 (8)	0.3811 (2)	0.0306 (10)	
S2_1	0.5454 (4)	0.09810 (9)	0.1811 (2)	0.0334 (10)	
Cl1_1	0.4126 (4)	0.04489 (9)	0.3510 (3)	0.0443 (11)	
Cl2_1	0.9536 (5)	0.12595 (10)	0.1865 (3)	0.0574 (14)	
O1_1	0.7636 (10)	0.0547 (2)	0.2521 (6)	0.036 (3)	
O2_1	0.5677 (13)	0.0163 (3)	0.2383 (7)	0.057 (4)	
O3_1	0.6895 (11)	0.0259 (2)	0.3765 (6)	0.040 (3)	
O4_1	1.0114 (11)	0.0698 (2)	0.2526 (7)	0.045 (3)	
O5_1	0.8667 (11)	0.0711 (2)	0.1205 (7)	0.048 (3)	
O6_1	0.6406 (10)	0.0817 (2)	0.3603 (6)	0.035 (3)	
O7_1	0.8371 (15)	0.1019 (3)	0.3224 (9)	0.079 (5)	
O8_1	0.6969 (12)	0.1276 (2)	0.3937 (8)	0.054 (4)	
O9_1	0.8211 (15)	0.0913 (3)	0.4486 (9)	0.082 (5)	
O10_1	0.5174 (11)	0.0714 (3)	0.2139 (7)	0.047 (3)	
O11_1	0.6839 (12)	0.1017 (2)	0.1803 (9)	0.066 (5)	
O12_1	0.4878 (15)	0.0989 (3)	0.1043 (7)	0.076 (5)	
O13_1	0.4953 (12)	0.1195 (3)	0.2274 (7)	0.050 (3)	
Mo1_2	0.07651 (14)	0.04591 (3)	0.80064 (8)	0.0342 (4)	
Mo2_2	0.32790 (14)	0.08398 (3)	0.71361 (8)	0.0336 (4)	
Cl1_2	−0.1186 (4)	0.04937 (10)	0.8657 (3)	0.0476 (12)	
Cl2_2	0.4061 (5)	0.12807 (10)	0.6787 (3)	0.0558 (14)	
S1_2	0.2164 (4)	0.10380 (9)	0.8868 (2)	0.0352 (11)	
S2_2	0.0087 (4)	0.10255 (9)	0.6850 (2)	0.0371 (11)	
O1_2	0.2183 (10)	0.0576 (2)	0.7524 (6)	0.035 (3)	
O2_2	0.1451 (12)	0.0256 (2)	0.8698 (7)	0.051 (3)	
O3_2	0.0076 (13)	0.0240 (3)	0.7343 (8)	0.063 (4)	
O4_2	0.4680 (12)	0.0750 (3)	0.7637 (7)	0.051 (3)	
O5_2	0.3428 (12)	0.0710 (2)	0.6265 (7)	0.047 (3)	
O6_2	0.1268 (14)	0.0807 (3)	0.8811 (8)	0.064 (4)	
O7_2	0.2701 (12)	0.1091 (2)	0.8123 (7)	0.048 (3)	
O8_2	0.3146 (14)	0.0977 (4)	0.9422 (10)	0.106 (7)	
O9_2	0.1473 (14)	0.1273 (2)	0.9043 (8)	0.063 (4)	
O10_2	−0.0181 (13)	0.0806 (3)	0.7395 (8)	0.073 (5)	
O11_2	0.1387 (13)	0.1019 (3)	0.6654 (8)	0.060 (4)	
O12_2	−0.0767 (16)	0.0992 (3)	0.6156 (9)	0.088 (5)	
O13_2	−0.0172 (15)	0.1276 (3)	0.7225 (10)	0.086 (5)	
Mo1_3	0.44358 (17)	0.23070 (4)	0.36070 (10)	0.0491 (5)	
Mo2_3	0.30556 (19)	0.29740 (4)	0.40168 (11)	0.0559 (5)	
Cl1_3	0.4093 (5)	0.18173 (10)	0.3646 (3)	0.0566 (13)	
Cl2_3	0.1181 (6)	0.31877 (11)	0.4444 (4)	0.0815 (19)	
S1_3	0.3402 (5)	0.24730 (9)	0.5328 (3)	0.0431 (12)	
S2_3	0.1355 (5)	0.24752 (9)	0.3051 (3)	0.0404 (12)	
O1_3	0.4123 (11)	0.2697 (3)	0.3742 (7)	0.057 (4)	
O2_3	0.5916 (13)	0.2273 (3)	0.4055 (9)	0.073 (4)	
O3_3	0.4727 (14)	0.2309 (3)	0.2679 (8)	0.071 (4)	
O4_3	0.3178 (19)	0.3169 (3)	0.3226 (9)	0.091 (5)	
O5_3	0.4034 (14)	0.3135 (3)	0.4681 (8)	0.068 (4)	
O6_3	0.3605 (12)	0.2252 (2)	0.4743 (7)	0.051 (3)	
O7_3	0.2559 (12)	0.2679 (3)	0.4923 (8)	0.058 (4)	
O8_3	0.4594 (13)	0.2594 (3)	0.5629 (8)	0.064 (4)	
O9_3	0.2722 (13)	0.2355 (3)	0.5928 (7)	0.057 (4)	
O10_3	0.2360 (12)	0.2280 (2)	0.3327 (8)	0.053 (3)	
O11_3	0.1574 (17)	0.2735 (3)	0.3401 (12)	0.115 (7)	
O12_3	0.138 (2)	0.2519 (5)	0.2268 (9)	0.139 (10)	
O13_3	0.0161 (17)	0.2380 (3)	0.3250 (17)	0.140 (10)	
N1_4	0.4861 (17)	0.0489 (4)	0.9445 (10)	0.063 (5)*	
H1_4	0.4257	0.0608	0.9318	0.076*	
C1_4	0.499 (2)	0.0285 (4)	0.8951 (13)	0.060 (6)*	
H1A_4	0.4482	0.0271	0.8479	0.073*	
C2_4	0.591 (2)	0.0100 (5)	0.9170 (13)	0.064 (6)*	
H2_4	0.6036	−0.0047	0.8837	0.077*	
C3_4	0.660 (2)	0.0114 (4)	0.9796 (12)	0.060 (6)*	
H3_4	0.7233	−0.0020	0.9930	0.072*	
C4_4	0.642 (2)	0.0336 (5)	1.0296 (14)	0.074 (7)*	
H4_4	0.6924	0.0350	1.0772	0.089*	
C5_4	0.5530 (17)	0.0527 (4)	1.0089 (11)	0.045 (5)*	
H5_4	0.5404	0.0680	1.0401	0.054*	
N1_5	0.6472 (17)	0.0571 (4)	0.5494 (11)	0.067 (5)*	
H1_5	0.6586	0.0652	0.5059	0.081*	
C1_5	0.5530 (19)	0.0387 (4)	0.5522 (12)	0.055 (5)*	
H1A_5	0.4969	0.0352	0.5078	0.066*	
C2_5	0.538 (2)	0.0258 (5)	0.6147 (13)	0.064 (6)*	
H2_5	0.4750	0.0121	0.6168	0.077*	
C3_5	0.6154 (18)	0.0324 (4)	0.6763 (11)	0.050 (5)*	
H3_5	0.6038	0.0239	0.7239	0.060*	
C4_5	0.709 (2)	0.0506 (4)	0.6724 (13)	0.062 (6)*	
H4_5	0.7666	0.0546	0.7157	0.074*	
C5_5	0.7179 (16)	0.0627 (3)	0.6075 (9)	0.034 (4)*	
H5_5	0.7811	0.0763	0.6044	0.041*	
N1_6	0.0519 (17)	0.0486 (4)	0.5239 (11)	0.069 (5)*	
H1_6	0.0376	0.0610	0.5583	0.083*	
C1_6	−0.0135 (16)	0.0490 (4)	0.4585 (10)	0.037 (4)*	
H1A_6	−0.0810	0.0617	0.4497	0.045*	
C2_6	0.0112 (19)	0.0317 (4)	0.4019 (12)	0.052 (5)*	
H2_6	−0.0365	0.0329	0.3535	0.063*	
C3_6	0.1016 (19)	0.0132 (4)	0.4137 (12)	0.056 (5)*	
H3_6	0.1176	0.0012	0.3733	0.067*	
C4_6	0.172 (2)	0.0108 (4)	0.4814 (11)	0.055 (5)*	
H4_6	0.2372	−0.0024	0.4891	0.066*	
C5_6	0.1446 (19)	0.0289 (4)	0.5410 (12)	0.057 (5)*	
H5_6	0.1883	0.0276	0.5905	0.068*	
N1_7	0.1357 (15)	0.0553 (3)	0.0363 (10)	0.054 (4)*	
H1_7	0.1290	0.0626	−0.0097	0.065*	
C1_7	0.056 (2)	0.0368 (5)	0.0525 (14)	0.070 (6)*	
H1A_7	−0.0124	0.0322	0.0157	0.084*	
C2_7	0.066 (2)	0.0240 (5)	0.1185 (13)	0.065 (6)*	
H2_7	0.0045	0.0105	0.1274	0.078*	
C3_7	0.1505 (18)	0.0288 (4)	0.1691 (12)	0.051 (5)*	
H3_7	0.1564	0.0191	0.2159	0.061*	
C4_7	0.248 (2)	0.0514 (4)	0.1543 (12)	0.056 (5)*	
H4_7	0.3169	0.0562	0.1902	0.068*	
C5_7	0.2252 (17)	0.0634 (4)	0.0859 (10)	0.043 (5)*	
H5_7	0.2768	0.0783	0.0737	0.052*	
N1_8	0.1397 (16)	0.1897 (4)	0.4548 (10)	0.063 (5)*	
H1_8	0.1876	0.2036	0.4441	0.076*	
C1_8	0.068 (2)	0.1803 (5)	0.3990 (15)	0.080 (7)*	
H1A_8	0.0713	0.1874	0.3489	0.096*	
C2_8	−0.018 (2)	0.1591 (5)	0.4134 (14)	0.074 (7)*	
H2_8	−0.0745	0.1515	0.3741	0.089*	
C3_8	−0.013 (2)	0.1502 (5)	0.4901 (13)	0.069 (6)*	
H3_8	−0.0684	0.1360	0.5028	0.083*	
C4_8	0.069 (2)	0.1611 (5)	0.5462 (15)	0.080 (7)*	
H4_8	0.0685	0.1551	0.5976	0.095*	
C5_8	0.152 (2)	0.1814 (5)	0.5269 (14)	0.073 (7)*	
H5_8	0.2146	0.1889	0.5634	0.088*	
N1_9	0.210 (2)	0.1652 (5)	0.7840 (12)	0.085 (6)*	
H1_9	0.2068	0.1474	0.7873	0.102*	
C1_9	0.115 (3)	0.1785 (7)	0.7720 (17)	0.100 (9)*	
H1A_9	0.0343	0.1698	0.7702	0.121*	
C2_9	0.120 (4)	0.2048 (8)	0.7612 (19)	0.121 (11)*	
H2_9	0.0408	0.2137	0.7480	0.145*	
C3_9	0.216 (3)	0.2193 (7)	0.7665 (17)	0.099 (9)*	
H3_9	0.2092	0.2386	0.7654	0.119*	
C4_9	0.331 (3)	0.2069 (7)	0.7738 (18)	0.107 (9)*	
H4_9	0.4084	0.2162	0.7670	0.128*	
C5_9	0.327 (3)	0.1789 (7)	0.7925 (19)	0.121 (11)*	
H5_9	0.4029	0.1696	0.8106	0.145*	
N1_10	0.1566 (18)	0.3143 (4)	0.1724 (11)	0.075 (5)*	
H1_10	0.1694	0.3076	0.2189	0.090*	
C1_10	0.222 (2)	0.3370 (4)	0.1531 (12)	0.060 (6)*	
H1A_10	0.2831	0.3453	0.1887	0.072*	
C2_10	0.1964 (19)	0.3475 (4)	0.0811 (12)	0.054 (5)*	
H2_10	0.2414	0.3629	0.0651	0.065*	
C3_10	0.111 (2)	0.3361 (5)	0.0356 (14)	0.065 (6)*	
H3_10	0.0878	0.3439	−0.0131	0.078*	
C4_10	0.0536 (18)	0.3137 (4)	0.0550 (11)	0.051 (5)*	
H4_10	−0.0058	0.3055	0.0182	0.061*	
C5_10	0.0732 (19)	0.3023 (4)	0.1207 (12)	0.055 (5)*	
H5_10	0.0305	0.2860	0.1323	0.066*	
N1_11	0.6750 (16)	0.1625 (4)	0.2403 (10)	0.060 (5)*	
H1_11	0.6374	0.1473	0.2516	0.072*	
C1_11	0.7116 (19)	0.1792 (4)	0.2946 (12)	0.054 (5)*	
H1A_11	0.6951	0.1755	0.3458	0.065*	
C2_11	0.775 (2)	0.2026 (5)	0.2768 (14)	0.073 (7)*	
H2_11	0.8020	0.2150	0.3162	0.087*	
C3_11	0.801 (2)	0.2082 (5)	0.2014 (13)	0.072 (7)*	
H3_11	0.8480	0.2238	0.1900	0.087*	
C4_11	0.756 (2)	0.1903 (5)	0.1433 (15)	0.075 (7)*	
H4_11	0.7673	0.1935	0.0910	0.090*	
C5_11	0.693 (2)	0.1678 (5)	0.1682 (13)	0.062 (6)*	
H5_11	0.6602	0.1552	0.1306	0.074*	
N1_12	0.599 (3)	0.3141 (6)	0.5825 (17)	0.118 (8)*	
H1_12	0.5301	0.3068	0.5582	0.142*	
C1_12	0.640 (3)	0.3049 (7)	0.645 (2)	0.115 (10)*	
H1A_12	0.6022	0.2894	0.6656	0.137*	
C2_12	0.733 (3)	0.3162 (7)	0.680 (2)	0.109 (10)*	
H2_12	0.7612	0.3101	0.7301	0.131*	
C3_12	0.799 (4)	0.3380 (8)	0.648 (2)	0.133 (12)*	
H3_12	0.8691	0.3465	0.6771	0.159*	
C4_12	0.760 (3)	0.3468 (6)	0.5751 (17)	0.089 (8)*	
H4_12	0.8046	0.3597	0.5472	0.107*	
C5_12	0.657 (3)	0.3355 (6)	0.5505 (18)	0.095 (8)*	
H5_12	0.6154	0.3426	0.5045	0.114*	
N1_13	0.3595 (14)	0.1116 (3)	0.3505 (9)	0.042 (4)*	
H1_13	0.4088	0.1138	0.3130	0.050*	
C1_13	0.2354 (19)	0.1070 (4)	0.3325 (12)	0.055 (5)*	
H1A_13	0.2010	0.1064	0.2807	0.066*	
C2_13	0.158 (2)	0.1030 (4)	0.3919 (12)	0.057 (5)*	
H2_13	0.0688	0.0998	0.3814	0.068*	
C3_13	0.2134 (18)	0.1037 (4)	0.4668 (11)	0.047 (5)*	
H3_13	0.1637	0.1004	0.5084	0.056*	
C4_13	0.341 (2)	0.1093 (4)	0.4794 (13)	0.059 (6)*	
H4_13	0.3803	0.1106	0.5300	0.071*	
C5_13	0.4102 (19)	0.1130 (4)	0.4182 (11)	0.050 (5)*	
H5_13	0.4989	0.1167	0.4268	0.060*	
N1_14	0.749 (3)	0.2531 (6)	0.5500 (17)	0.130 (9)*	
H1_14	0.6683	0.2507	0.5579	0.156*	
C1_14	0.853 (3)	0.2483 (6)	0.6095 (18)	0.096 (9)*	
H1A_14	0.8350	0.2424	0.6589	0.115*	
C2_14	0.987 (3)	0.2527 (6)	0.5917 (19)	0.110 (10)*	
H2_14	1.0590	0.2503	0.6279	0.132*	
C3_14	0.995 (2)	0.2603 (4)	0.5196 (13)	0.059 (6)*	
H3_14	1.0788	0.2638	0.5054	0.071*	
C4_14	0.906 (4)	0.2633 (7)	0.468 (2)	0.129 (12)*	
H4_14	0.9305	0.2667	0.4183	0.155*	
C5_14	0.791 (4)	0.2623 (8)	0.475 (2)	0.148 (14)*	
H5_14	0.7301	0.2672	0.4335	0.177*	
N1_15	0.3633 (19)	0.3831 (4)	0.3870 (11)	0.071 (5)*	
H1_15	0.4409	0.3830	0.3719	0.085*	
C1_15	0.161 (2)	0.3844 (5)	0.3563 (14)	0.074 (7)*	
H1A_15	0.0926	0.3843	0.3173	0.088*	
C2_15	0.136 (2)	0.3864 (5)	0.4289 (15)	0.078 (7)*	
H2_15	0.0502	0.3878	0.4415	0.093*	
C3_15	0.236 (2)	0.3863 (4)	0.4865 (13)	0.059 (6)*	
H3_15	0.2240	0.3880	0.5392	0.071*	
C4_15	0.351 (3)	0.3837 (5)	0.4608 (17)	0.090 (8)*	
H4_15	0.4241	0.3823	0.4962	0.108*	
C5_15	0.273 (2)	0.3828 (5)	0.3384 (15)	0.077 (7)*	
H5_15	0.2876	0.3812	0.2860	0.092*	

### Atomic displacement parameters (Å^2^)

**Table:**

	*U*^11^	*U*^22^	*U*^33^	*U*^12^	*U*^13^	*U*^23^
Mo1_1	0.0431 (9)	0.0243 (8)	0.0317 (9)	−0.0068 (7)	0.0027 (7)	−0.0008 (7)
Mo2_1	0.0344 (9)	0.0332 (9)	0.0371 (9)	0.0008 (7)	0.0092 (7)	−0.0010 (7)
S1_1	0.037 (2)	0.024 (2)	0.030 (2)	−0.006 (2)	0.0001 (19)	−0.0027 (18)
S2_1	0.034 (3)	0.040 (3)	0.026 (2)	−0.006 (2)	0.0014 (19)	0.000 (2)
Cl1_1	0.043 (3)	0.040 (3)	0.050 (3)	−0.008 (2)	0.001 (2)	0.005 (2)
Cl2_1	0.041 (3)	0.049 (3)	0.082 (4)	−0.007 (2)	0.009 (3)	0.010 (3)
O1_1	0.041 (7)	0.034 (7)	0.033 (6)	−0.008 (5)	0.012 (5)	−0.002 (5)
O2_1	0.082 (10)	0.041 (8)	0.048 (8)	−0.016 (7)	0.007 (7)	−0.007 (6)
O3_1	0.044 (7)	0.030 (7)	0.045 (7)	−0.003 (6)	−0.007 (6)	0.012 (5)
O4_1	0.038 (7)	0.047 (8)	0.051 (8)	−0.003 (6)	0.007 (6)	0.003 (6)
O5_1	0.048 (8)	0.045 (8)	0.050 (8)	−0.001 (6)	0.011 (6)	−0.008 (6)
O6_1	0.037 (7)	0.034 (7)	0.036 (7)	−0.001 (6)	0.010 (5)	−0.008 (5)
O7_1	0.084 (11)	0.073 (10)	0.088 (11)	−0.046 (9)	0.054 (9)	−0.047 (9)
O8_1	0.053 (8)	0.013 (6)	0.098 (11)	0.011 (6)	0.014 (7)	−0.012 (6)
O9_1	0.092 (11)	0.055 (10)	0.090 (12)	−0.021 (8)	−0.052 (9)	0.023 (8)
O10_1	0.039 (7)	0.055 (8)	0.044 (8)	−0.017 (6)	−0.014 (6)	0.028 (6)
O11_1	0.038 (8)	0.025 (7)	0.140 (14)	0.013 (6)	0.038 (8)	0.031 (8)
O12_1	0.101 (12)	0.098 (12)	0.024 (8)	−0.031 (10)	−0.021 (7)	0.014 (7)
O13_1	0.056 (8)	0.049 (8)	0.047 (8)	0.019 (7)	0.020 (7)	0.002 (6)
Mo1_2	0.0411 (9)	0.0275 (9)	0.0340 (9)	−0.0055 (7)	0.0037 (7)	−0.0003 (7)
Mo2_2	0.0370 (9)	0.0321 (9)	0.0327 (9)	−0.0024 (7)	0.0083 (7)	−0.0038 (7)
Cl1_2	0.044 (3)	0.060 (3)	0.040 (3)	−0.007 (2)	0.006 (2)	0.009 (2)
Cl2_2	0.080 (4)	0.040 (3)	0.052 (3)	−0.012 (3)	0.030 (3)	−0.004 (2)
S1_2	0.038 (3)	0.038 (3)	0.030 (2)	−0.010 (2)	0.004 (2)	0.001 (2)
S2_2	0.043 (3)	0.038 (3)	0.029 (3)	0.007 (2)	−0.003 (2)	0.008 (2)
O1_2	0.035 (7)	0.031 (7)	0.041 (7)	−0.002 (5)	0.012 (5)	−0.002 (5)
O2_2	0.064 (9)	0.033 (7)	0.057 (9)	0.006 (6)	0.015 (7)	0.017 (6)
O3_2	0.065 (9)	0.062 (9)	0.064 (9)	−0.039 (8)	0.016 (7)	−0.039 (7)
O4_2	0.050 (8)	0.058 (9)	0.044 (8)	−0.012 (7)	−0.004 (6)	0.001 (6)
O5_2	0.055 (8)	0.048 (8)	0.039 (7)	−0.007 (6)	0.016 (6)	−0.015 (6)
O6_2	0.095 (11)	0.045 (8)	0.056 (9)	−0.040 (8)	0.031 (8)	−0.010 (7)
O7_2	0.073 (9)	0.034 (7)	0.042 (8)	−0.015 (6)	0.025 (7)	−0.010 (6)
O8_2	0.054 (10)	0.169 (19)	0.090 (13)	−0.016 (11)	−0.021 (9)	0.067 (13)
O9_2	0.081 (10)	0.031 (8)	0.087 (11)	−0.009 (7)	0.053 (9)	−0.009 (7)
O10_2	0.053 (9)	0.092 (12)	0.079 (10)	0.021 (8)	0.030 (8)	0.066 (9)
O11_2	0.068 (10)	0.055 (9)	0.060 (9)	0.018 (7)	0.026 (7)	0.024 (7)
O12_2	0.108 (13)	0.083 (12)	0.068 (11)	−0.063 (10)	−0.014 (10)	0.008 (9)
O13_2	0.079 (11)	0.060 (10)	0.122 (14)	0.015 (8)	0.018 (10)	−0.047 (10)
Mo1_3	0.0509 (11)	0.0491 (11)	0.0480 (11)	0.0003 (9)	0.0093 (8)	0.0008 (8)
Mo2_3	0.0731 (13)	0.0329 (10)	0.0597 (12)	−0.0047 (9)	−0.0048 (10)	−0.0003 (9)
Cl1_3	0.075 (3)	0.040 (3)	0.054 (3)	0.013 (3)	0.003 (3)	−0.008 (2)
Cl2_3	0.084 (4)	0.041 (3)	0.116 (5)	0.006 (3)	−0.011 (4)	−0.006 (3)
S1_3	0.043 (3)	0.031 (3)	0.057 (3)	0.003 (2)	0.015 (3)	−0.006 (2)
S2_3	0.051 (3)	0.029 (2)	0.042 (3)	−0.001 (2)	0.008 (2)	−0.002 (2)
O1_3	0.040 (7)	0.086 (11)	0.044 (8)	0.000 (7)	−0.005 (6)	−0.027 (7)
O2_3	0.055 (9)	0.079 (11)	0.086 (11)	0.000 (8)	0.020 (8)	−0.016 (9)
O3_3	0.084 (11)	0.074 (11)	0.060 (10)	−0.003 (9)	0.031 (8)	0.004 (8)
O4_3	0.156 (16)	0.061 (11)	0.057 (10)	0.029 (10)	0.012 (10)	0.008 (8)
O5_3	0.086 (10)	0.044 (9)	0.072 (10)	−0.020 (8)	−0.007 (8)	−0.011 (7)
O6_3	0.055 (8)	0.044 (8)	0.056 (8)	−0.011 (6)	0.020 (7)	−0.008 (6)
O7_3	0.050 (8)	0.049 (9)	0.076 (10)	0.018 (7)	0.011 (7)	0.019 (7)
O8_3	0.053 (9)	0.077 (11)	0.061 (9)	−0.004 (8)	0.004 (7)	−0.018 (8)
O9_3	0.081 (10)	0.047 (8)	0.047 (8)	0.002 (7)	0.029 (7)	−0.011 (6)
O10_3	0.056 (8)	0.025 (7)	0.077 (10)	−0.004 (6)	0.000 (7)	0.011 (6)
O11_3	0.113 (14)	0.046 (10)	0.170 (19)	0.027 (9)	−0.080 (13)	−0.055 (11)
O12_3	0.21 (2)	0.17 (2)	0.046 (10)	0.114 (18)	0.017 (12)	0.048 (12)
O13_3	0.071 (12)	0.052 (11)	0.31 (3)	0.007 (9)	0.089 (16)	0.047 (15)

### Geometric parameters (Å, °)

**Table:**

Mo1_1—O3_1	1.711 (11)	C2_7—H2_7	0.9500
Mo1_1—O2_1	1.717 (12)	C3_7—C4_7	1.55 (3)
Mo1_1—O1_1	1.891 (10)	C3_7—H3_7	0.9500
Mo1_1—O6_1	2.224 (11)	C4_7—C5_7	1.34 (3)
Mo1_1—O10_1	2.255 (11)	C4_7—H4_7	0.9500
Mo1_1—Cl1_1	2.431 (5)	C5_7—H5_7	0.9500
Mo2_1—O4_1	1.690 (12)	N1_8—C1_8	1.27 (3)
Mo2_1—O5_1	1.705 (12)	N1_8—C5_8	1.33 (3)
Mo2_1—O1_1	1.893 (10)	N1_8—H1_8	0.8800
Mo2_1—O7_1	2.217 (13)	C1_8—C2_8	1.41 (3)
Mo2_1—O11_1	2.222 (12)	C1_8—H1A_8	0.9500
Mo2_1—Cl2_1	2.424 (5)	C2_8—C3_8	1.41 (3)
S1_1—O9_1	1.434 (15)	C2_8—H2_8	0.9500
S1_1—O8_1	1.447 (11)	C3_8—C4_8	1.36 (3)
S1_1—O7_1	1.457 (12)	C3_8—H3_8	0.9500
S1_1—O6_1	1.491 (12)	C4_8—C5_8	1.39 (3)
S2_1—O12_1	1.430 (14)	C4_8—H4_8	0.9500
S2_1—O13_1	1.456 (12)	C5_8—H5_8	0.9500
S2_1—O11_1	1.469 (13)	N1_9—C1_9	1.20 (3)
S2_1—O10_1	1.478 (12)	N1_9—C5_9	1.40 (4)
Mo1_2—O2_2	1.684 (12)	N1_9—H1_9	0.8800
Mo1_2—O3_2	1.701 (12)	C1_9—C2_9	1.31 (4)
Mo1_2—O1_2	1.870 (10)	C1_9—H1A_9	0.9500
Mo1_2—O10_2	2.210 (13)	C2_9—C3_9	1.23 (4)
Mo1_2—O6_2	2.252 (13)	C2_9—H2_9	0.9500
Mo1_2—Cl1_2	2.442 (5)	C3_9—C4_9	1.35 (4)
Mo2_2—O5_2	1.678 (11)	C3_9—H3_9	0.9500
Mo2_2—O4_2	1.706 (13)	C4_9—C5_9	1.42 (4)
Mo2_2—O1_2	1.902 (10)	C4_9—H4_9	0.9500
Mo2_2—O7_2	2.257 (11)	C5_9—H5_9	0.9500
Mo2_2—O11_2	2.271 (14)	N1_10—C5_10	1.34 (3)
Mo2_2—Cl2_2	2.421 (5)	N1_10—C1_10	1.37 (3)
S1_2—O8_2	1.387 (16)	N1_10—H1_10	0.8800
S1_2—O9_2	1.417 (13)	C1_10—C2_10	1.37 (3)
S1_2—O6_2	1.477 (13)	C1_10—H1A_10	0.9500
S1_2—O7_2	1.494 (11)	C2_10—C3_10	1.28 (3)
S2_2—O13_2	1.435 (14)	C2_10—H2_10	0.9500
S2_2—O11_2	1.439 (13)	C3_10—C4_10	1.31 (3)
S2_2—O12_2	1.458 (16)	C3_10—H3_10	0.9500
S2_2—O10_2	1.485 (12)	C4_10—C5_10	1.28 (3)
Mo1_3—O3_3	1.684 (13)	C4_10—H4_10	0.9500
Mo1_3—O2_3	1.689 (16)	C5_10—H5_10	0.9500
Mo1_3—O1_3	1.969 (14)	N1_11—C1_11	1.29 (2)
Mo1_3—O10_3	2.200 (13)	N1_11—C5_11	1.32 (2)
Mo1_3—O6_3	2.264 (12)	N1_11—H1_11	0.8800
Mo1_3—Cl1_3	2.442 (5)	C1_11—C2_11	1.38 (3)
Mo2_3—O5_3	1.684 (14)	C1_11—H1A_11	0.9500
Mo2_3—O4_3	1.704 (15)	C2_11—C3_11	1.40 (3)
Mo2_3—O1_3	1.857 (13)	C2_11—H2_11	0.9500
Mo2_3—O11_3	2.164 (16)	C3_11—C4_11	1.40 (3)
Mo2_3—O7_3	2.249 (12)	C3_11—H3_11	0.9500
Mo2_3—Cl2_3	2.413 (7)	C4_11—C5_11	1.38 (3)
S1_3—O8_3	1.445 (15)	C4_11—H4_11	0.9500
S1_3—O9_3	1.448 (12)	C5_11—H5_11	0.9500
S1_3—O7_3	1.488 (13)	N1_12—C1_12	1.23 (4)
S1_3—O6_3	1.522 (13)	N1_12—C5_12	1.37 (3)
S2_3—O12_3	1.395 (16)	N1_12—H1_12	0.8800
S2_3—O13_3	1.412 (16)	C1_12—C2_12	1.24 (4)
S2_3—O11_3	1.431 (15)	C1_12—H1A_12	0.9500
S2_3—O10_3	1.480 (13)	C2_12—C3_12	1.42 (4)
N1_4—C5_4	1.29 (2)	C2_12—H2_12	0.9500
N1_4—C1_4	1.34 (2)	C3_12—C4_12	1.39 (4)
N1_4—H1_4	0.8800	C3_12—H3_12	0.9500
C1_4—C2_4	1.36 (3)	C4_12—C5_12	1.26 (4)
C1_4—H1A_4	0.9500	C4_12—H4_12	0.9500
C2_4—C3_4	1.27 (3)	C5_12—H5_12	0.9500
C2_4—H2_4	0.9500	N1_13—C5_13	1.26 (2)
C3_4—C4_4	1.43 (3)	N1_13—C1_13	1.33 (2)
C3_4—H3_4	0.9500	N1_13—H1_13	0.8800
C4_4—C5_4	1.35 (3)	C1_13—C2_13	1.39 (2)
C4_4—H4_4	0.9500	C1_13—H1A_13	0.9500
C5_4—H5_4	0.9500	C2_13—C3_13	1.39 (3)
N1_5—C5_5	1.24 (2)	C2_13—H2_13	0.9500
N1_5—C1_5	1.35 (2)	C3_13—C4_13	1.37 (3)
N1_5—H1_5	0.8800	C3_13—H3_13	0.9500
C1_5—C2_5	1.29 (3)	C4_13—C5_13	1.36 (2)
C1_5—H1A_5	0.9500	C4_13—H4_13	0.9500
C2_5—C3_5	1.33 (3)	C5_13—H5_13	0.9500
C2_5—H2_5	0.9500	N1_14—C1_14	1.46 (4)
C3_5—C4_5	1.34 (3)	N1_14—C5_14	1.50 (4)
C3_5—H3_5	0.9500	N1_14—H1_14	0.8800
C4_5—C5_5	1.30 (2)	C1_14—C2_14	1.49 (4)
C4_5—H4_5	0.9500	C1_14—H1A_14	0.9500
C5_5—H5_5	0.9500	C2_14—C3_14	1.33 (3)
N1_6—C1_6	1.29 (2)	C2_14—H2_14	0.9500
N1_6—C5_6	1.39 (3)	C3_14—C4_14	1.25 (4)
N1_6—H1_6	0.8800	C3_14—H3_14	0.9500
C1_6—C2_6	1.35 (2)	C4_14—C5_14	1.23 (4)
C1_6—H1A_6	0.9500	C4_14—H4_14	0.9500
C2_6—C3_6	1.32 (3)	C5_14—H5_14	0.9500
C2_6—H2_6	0.9500	N1_15—C5_15	1.22 (3)
C3_6—C4_6	1.35 (3)	N1_15—C4_15	1.32 (3)
C3_6—H3_6	0.9500	N1_15—H1_15	0.8800
C4_6—C5_6	1.42 (3)	C1_15—C5_15	1.24 (3)
C4_6—H4_6	0.9500	C1_15—C2_15	1.33 (3)
C5_6—H5_6	0.9500	C1_15—H1A_15	0.9500
N1_7—C5_7	1.29 (2)	C2_15—C3_15	1.40 (3)
N1_7—C1_7	1.29 (2)	C2_15—H2_15	0.9500
N1_7—H1_7	0.8800	C3_15—C4_15	1.33 (3)
C1_7—C2_7	1.31 (3)	C3_15—H3_15	0.9500
C1_7—H1A_7	0.9500	C4_15—H4_15	0.9500
C2_7—C3_7	1.22 (3)	C5_15—H5_15	0.9500
			
O3_1—Mo1_1—O2_1	103.1 (6)	C5_6—N1_6—H1_6	119.6
O3_1—Mo1_1—O1_1	100.7 (5)	N1_6—C1_6—C2_6	121.7 (19)
O2_1—Mo1_1—O1_1	101.3 (5)	N1_6—C1_6—H1A_6	119.1
O3_1—Mo1_1—O6_1	90.2 (5)	C2_6—C1_6—H1A_6	119.1
O2_1—Mo1_1—O6_1	165.0 (6)	C3_6—C2_6—C1_6	120 (2)
O1_1—Mo1_1—O6_1	82.9 (4)	C3_6—C2_6—H2_6	119.9
O3_1—Mo1_1—O10_1	167.5 (5)	C1_6—C2_6—H2_6	119.9
O2_1—Mo1_1—O10_1	88.1 (6)	C2_6—C3_6—C4_6	122 (2)
O1_1—Mo1_1—O10_1	82.3 (4)	C2_6—C3_6—H3_6	119.1
O6_1—Mo1_1—O10_1	78.1 (4)	C4_6—C3_6—H3_6	119.1
O3_1—Mo1_1—Cl1_1	93.4 (4)	C3_6—C4_6—C5_6	118 (2)
O2_1—Mo1_1—Cl1_1	91.9 (5)	C3_6—C4_6—H4_6	121.2
O1_1—Mo1_1—Cl1_1	158.0 (4)	C5_6—C4_6—H4_6	121.2
O6_1—Mo1_1—Cl1_1	80.1 (3)	N1_6—C5_6—C4_6	118 (2)
O10_1—Mo1_1—Cl1_1	80.5 (3)	N1_6—C5_6—H5_6	121.2
O4_1—Mo2_1—O5_1	103.2 (6)	C4_6—C5_6—H5_6	121.2
O4_1—Mo2_1—O1_1	98.1 (5)	C5_7—N1_7—C1_7	121.4 (19)
O5_1—Mo2_1—O1_1	100.0 (5)	C5_7—N1_7—H1_7	119.3
O4_1—Mo2_1—O7_1	89.9 (6)	C1_7—N1_7—H1_7	119.3
O5_1—Mo2_1—O7_1	166.2 (7)	N1_7—C1_7—C2_7	122 (2)
O1_1—Mo2_1—O7_1	82.1 (5)	N1_7—C1_7—H1A_7	119.0
O4_1—Mo2_1—O11_1	169.4 (6)	C2_7—C1_7—H1A_7	119.0
O5_1—Mo2_1—O11_1	86.9 (6)	C3_7—C2_7—C1_7	123 (2)
O1_1—Mo2_1—O11_1	82.8 (5)	C3_7—C2_7—H2_7	118.6
O7_1—Mo2_1—O11_1	79.8 (6)	C1_7—C2_7—H2_7	118.6
O4_1—Mo2_1—Cl2_1	93.6 (4)	C2_7—C3_7—C4_7	118 (2)
O5_1—Mo2_1—Cl2_1	93.6 (4)	C2_7—C3_7—H3_7	121.1
O1_1—Mo2_1—Cl2_1	159.4 (3)	C4_7—C3_7—H3_7	121.1
O7_1—Mo2_1—Cl2_1	81.0 (4)	C5_7—C4_7—C3_7	113.6 (19)
O11_1—Mo2_1—Cl2_1	82.6 (3)	C5_7—C4_7—H4_7	123.2
O9_1—S1_1—O8_1	110.7 (9)	C3_7—C4_7—H4_7	123.2
O9_1—S1_1—O7_1	105.3 (11)	N1_7—C5_7—C4_7	122.0 (19)
O8_1—S1_1—O7_1	110.3 (8)	N1_7—C5_7—H5_7	119.0
O9_1—S1_1—O6_1	109.6 (8)	C4_7—C5_7—H5_7	119.0
O8_1—S1_1—O6_1	109.4 (7)	C1_8—N1_8—C5_8	129 (2)
O7_1—S1_1—O6_1	111.6 (7)	C1_8—N1_8—H1_8	115.7
O12_1—S2_1—O13_1	111.2 (9)	C5_8—N1_8—H1_8	115.7
O12_1—S2_1—O11_1	109.0 (9)	N1_8—C1_8—C2_8	118 (2)
O13_1—S2_1—O11_1	109.1 (8)	N1_8—C1_8—H1A_8	120.8
O12_1—S2_1—O10_1	108.0 (8)	C2_8—C1_8—H1A_8	120.8
O13_1—S2_1—O10_1	109.4 (7)	C3_8—C2_8—C1_8	115 (2)
O11_1—S2_1—O10_1	110.1 (7)	C3_8—C2_8—H2_8	122.3
Mo1_1—O1_1—Mo2_1	154.4 (6)	C1_8—C2_8—H2_8	122.3
S1_1—O6_1—Mo1_1	135.7 (6)	C4_8—C3_8—C2_8	122 (2)
S1_1—O7_1—Mo2_1	139.0 (8)	C4_8—C3_8—H3_8	118.9
S2_1—O10_1—Mo1_1	136.2 (7)	C2_8—C3_8—H3_8	118.9
S2_1—O11_1—Mo2_1	142.5 (7)	C3_8—C4_8—C5_8	119 (2)
O2_2—Mo1_2—O3_2	104.3 (7)	C3_8—C4_8—H4_8	120.7
O2_2—Mo1_2—O1_2	101.6 (5)	C5_8—C4_8—H4_8	120.7
O3_2—Mo1_2—O1_2	101.2 (5)	N1_8—C5_8—C4_8	116 (2)
O2_2—Mo1_2—O10_2	162.8 (6)	N1_8—C5_8—H5_8	121.9
O3_2—Mo1_2—O10_2	90.7 (7)	C4_8—C5_8—H5_8	121.9
O1_2—Mo1_2—O10_2	83.3 (5)	C1_9—N1_9—C5_9	118 (3)
O2_2—Mo1_2—O6_2	86.4 (6)	C1_9—N1_9—H1_9	121.2
O3_2—Mo1_2—O6_2	166.7 (7)	C5_9—N1_9—H1_9	121.2
O1_2—Mo1_2—O6_2	84.0 (5)	N1_9—C1_9—C2_9	122 (4)
O10_2—Mo1_2—O6_2	77.7 (6)	N1_9—C1_9—H1A_9	119.2
O2_2—Mo1_2—Cl1_2	91.6 (4)	C2_9—C1_9—H1A_9	119.2
O3_2—Mo1_2—Cl1_2	92.3 (4)	C3_9—C2_9—C1_9	127 (4)
O1_2—Mo1_2—Cl1_2	158.0 (4)	C3_9—C2_9—H2_9	116.4
O10_2—Mo1_2—Cl1_2	79.2 (3)	C1_9—C2_9—H2_9	116.4
O6_2—Mo1_2—Cl1_2	79.3 (3)	C2_9—C3_9—C4_9	118 (4)
O5_2—Mo2_2—O4_2	103.0 (6)	C2_9—C3_9—H3_9	121.2
O5_2—Mo2_2—O1_2	100.1 (5)	C4_9—C3_9—H3_9	121.2
O4_2—Mo2_2—O1_2	99.3 (5)	C3_9—C4_9—C5_9	115 (3)
O5_2—Mo2_2—O7_2	164.2 (6)	C3_9—C4_9—H4_9	122.7
O4_2—Mo2_2—O7_2	91.5 (6)	C5_9—C4_9—H4_9	122.7
O1_2—Mo2_2—O7_2	83.5 (4)	N1_9—C5_9—C4_9	119 (3)
O5_2—Mo2_2—O11_2	87.6 (6)	N1_9—C5_9—H5_9	120.3
O4_2—Mo2_2—O11_2	168.9 (6)	C4_9—C5_9—H5_9	120.3
O1_2—Mo2_2—O11_2	82.0 (5)	C5_10—N1_10—C1_10	120 (2)
O7_2—Mo2_2—O11_2	77.6 (5)	C5_10—N1_10—H1_10	119.9
O5_2—Mo2_2—Cl2_2	92.9 (4)	C1_10—N1_10—H1_10	119.9
O4_2—Mo2_2—Cl2_2	93.8 (4)	C2_10—C1_10—N1_10	118 (2)
O1_2—Mo2_2—Cl2_2	158.9 (3)	C2_10—C1_10—H1A_10	120.8
O7_2—Mo2_2—Cl2_2	79.7 (3)	N1_10—C1_10—H1A_10	120.8
O11_2—Mo2_2—Cl2_2	82.1 (4)	C3_10—C2_10—C1_10	119 (2)
O8_2—S1_2—O9_2	112.9 (12)	C3_10—C2_10—H2_10	120.7
O8_2—S1_2—O6_2	108.2 (10)	C1_10—C2_10—H2_10	120.7
O9_2—S1_2—O6_2	108.2 (8)	C2_10—C3_10—C4_10	121 (2)
O8_2—S1_2—O7_2	109.6 (9)	C2_10—C3_10—H3_10	119.4
O9_2—S1_2—O7_2	106.6 (8)	C4_10—C3_10—H3_10	119.4
O6_2—S1_2—O7_2	111.4 (7)	C5_10—C4_10—C3_10	124 (2)
O13_2—S2_2—O11_2	110.6 (9)	C5_10—C4_10—H4_10	118.0
O13_2—S2_2—O12_2	110.6 (11)	C3_10—C4_10—H4_10	118.0
O11_2—S2_2—O12_2	109.0 (9)	C4_10—C5_10—N1_10	117 (2)
O13_2—S2_2—O10_2	106.0 (10)	C4_10—C5_10—H5_10	121.4
O11_2—S2_2—O10_2	112.1 (7)	N1_10—C5_10—H5_10	121.4
O12_2—S2_2—O10_2	108.5 (9)	C1_11—N1_11—C5_11	121 (2)
Mo1_2—O1_2—Mo2_2	155.0 (6)	C1_11—N1_11—H1_11	119.3
S1_2—O6_2—Mo1_2	138.0 (7)	C5_11—N1_11—H1_11	119.3
S1_2—O7_2—Mo2_2	136.4 (7)	N1_11—C1_11—C2_11	119 (2)
S2_2—O10_2—Mo1_2	140.6 (8)	N1_11—C1_11—H1A_11	120.5
S2_2—O11_2—Mo2_2	137.6 (7)	C2_11—C1_11—H1A_11	120.5
O3_3—Mo1_3—O2_3	102.2 (7)	C1_11—C2_11—C3_11	121 (2)
O3_3—Mo1_3—O1_3	99.0 (6)	C1_11—C2_11—H2_11	119.5
O2_3—Mo1_3—O1_3	101.4 (6)	C3_11—C2_11—H2_11	119.5
O3_3—Mo1_3—O10_3	92.6 (7)	C4_11—C3_11—C2_11	119 (2)
O2_3—Mo1_3—O10_3	162.5 (6)	C4_11—C3_11—H3_11	120.5
O1_3—Mo1_3—O10_3	85.1 (5)	C2_11—C3_11—H3_11	120.5
O3_3—Mo1_3—O6_3	166.1 (6)	C5_11—C4_11—C3_11	115 (2)
O2_3—Mo1_3—O6_3	89.4 (6)	C5_11—C4_11—H4_11	122.7
O1_3—Mo1_3—O6_3	85.9 (5)	C3_11—C4_11—H4_11	122.7
O10_3—Mo1_3—O6_3	74.8 (5)	N1_11—C5_11—C4_11	125 (2)
O3_3—Mo1_3—Cl1_3	94.2 (5)	N1_11—C5_11—H5_11	117.5
O2_3—Mo1_3—Cl1_3	91.2 (5)	C4_11—C5_11—H5_11	117.5
O1_3—Mo1_3—Cl1_3	159.3 (4)	C1_12—N1_12—C5_12	121 (3)
O10_3—Mo1_3—Cl1_3	78.4 (3)	C1_12—N1_12—H1_12	119.6
O6_3—Mo1_3—Cl1_3	77.8 (3)	C5_12—N1_12—H1_12	119.6
O5_3—Mo2_3—O4_3	102.3 (8)	N1_12—C1_12—C2_12	119 (4)
O5_3—Mo2_3—O1_3	100.3 (6)	N1_12—C1_12—H1A_12	120.6
O4_3—Mo2_3—O1_3	96.4 (7)	C2_12—C1_12—H1A_12	120.6
O5_3—Mo2_3—O11_3	166.2 (8)	C1_12—C2_12—C3_12	122 (4)
O4_3—Mo2_3—O11_3	90.2 (9)	C1_12—C2_12—H2_12	118.8
O1_3—Mo2_3—O11_3	84.0 (6)	C3_12—C2_12—H2_12	118.8
O5_3—Mo2_3—O7_3	88.9 (6)	C4_12—C3_12—C2_12	119 (4)
O4_3—Mo2_3—O7_3	168.6 (8)	C4_12—C3_12—H3_12	120.5
O1_3—Mo2_3—O7_3	83.9 (5)	C2_12—C3_12—H3_12	120.5
O11_3—Mo2_3—O7_3	78.5 (7)	C5_12—C4_12—C3_12	111 (3)
O5_3—Mo2_3—Cl2_3	92.6 (5)	C5_12—C4_12—H4_12	124.6
O4_3—Mo2_3—Cl2_3	97.4 (6)	C3_12—C4_12—H4_12	124.6
O1_3—Mo2_3—Cl2_3	158.6 (4)	C4_12—C5_12—N1_12	127 (3)
O11_3—Mo2_3—Cl2_3	79.7 (5)	C4_12—C5_12—H5_12	116.4
O7_3—Mo2_3—Cl2_3	79.3 (3)	N1_12—C5_12—H5_12	116.4
O8_3—S1_3—O9_3	111.6 (9)	C5_13—N1_13—C1_13	123.7 (17)
O8_3—S1_3—O7_3	110.7 (9)	C5_13—N1_13—H1_13	118.1
O9_3—S1_3—O7_3	108.1 (8)	C1_13—N1_13—H1_13	118.1
O8_3—S1_3—O6_3	111.9 (8)	N1_13—C1_13—C2_13	118 (2)
O9_3—S1_3—O6_3	108.1 (7)	N1_13—C1_13—H1A_13	120.9
O7_3—S1_3—O6_3	106.2 (8)	C2_13—C1_13—H1A_13	120.9
O12_3—S2_3—O13_3	113.0 (17)	C3_13—C2_13—C1_13	118.5 (19)
O12_3—S2_3—O11_3	105.6 (15)	C3_13—C2_13—H2_13	120.7
O13_3—S2_3—O11_3	107.7 (13)	C1_13—C2_13—H2_13	120.7
O12_3—S2_3—O10_3	110.3 (10)	C4_13—C3_13—C2_13	118.9 (18)
O13_3—S2_3—O10_3	109.1 (9)	C4_13—C3_13—H3_13	120.6
O11_3—S2_3—O10_3	111.0 (9)	C2_13—C3_13—H3_13	120.6
Mo2_3—O1_3—Mo1_3	149.3 (7)	C5_13—C4_13—C3_13	119 (2)
S1_3—O6_3—Mo1_3	126.7 (7)	C5_13—C4_13—H4_13	120.5
S1_3—O7_3—Mo2_3	127.8 (7)	C3_13—C4_13—H4_13	120.5
S2_3—O10_3—Mo1_3	134.3 (7)	N1_13—C5_13—C4_13	122 (2)
S2_3—O11_3—Mo2_3	141.6 (10)	N1_13—C5_13—H5_13	119.2
C5_4—N1_4—C1_4	126 (2)	C4_13—C5_13—H5_13	119.2
C5_4—N1_4—H1_4	116.8	C1_14—N1_14—C5_14	115 (3)
C1_4—N1_4—H1_4	116.8	C1_14—N1_14—H1_14	122.5
N1_4—C1_4—C2_4	115 (2)	C5_14—N1_14—H1_14	122.5
N1_4—C1_4—H1A_4	122.3	N1_14—C1_14—C2_14	119 (3)
C2_4—C1_4—H1A_4	122.3	N1_14—C1_14—H1A_14	120.3
C3_4—C2_4—C1_4	123 (2)	C2_14—C1_14—H1A_14	120.3
C3_4—C2_4—H2_4	118.3	C3_14—C2_14—C1_14	112 (3)
C1_4—C2_4—H2_4	118.3	C3_14—C2_14—H2_14	123.8
C2_4—C3_4—C4_4	118 (2)	C1_14—C2_14—H2_14	123.8
C2_4—C3_4—H3_4	120.8	C4_14—C3_14—C2_14	128 (3)
C4_4—C3_4—H3_4	120.8	C4_14—C3_14—H3_14	116.1
C5_4—C4_4—C3_4	120 (2)	C2_14—C3_14—H3_14	116.1
C5_4—C4_4—H4_4	120.1	C5_14—C4_14—C3_14	128 (4)
C3_4—C4_4—H4_4	120.1	C5_14—C4_14—H4_14	116.1
N1_4—C5_4—C4_4	116 (2)	C3_14—C4_14—H4_14	116.1
N1_4—C5_4—H5_4	121.8	C4_14—C5_14—N1_14	117 (4)
C4_4—C5_4—H5_4	121.8	C4_14—C5_14—H5_14	121.4
C5_5—N1_5—C1_5	120.7 (19)	N1_14—C5_14—H5_14	121.4
C5_5—N1_5—H1_5	119.7	C5_15—N1_15—C4_15	123 (2)
C1_5—N1_5—H1_5	119.7	C5_15—N1_15—H1_15	118.4
C2_5—C1_5—N1_5	120 (2)	C4_15—N1_15—H1_15	118.4
C2_5—C1_5—H1A_5	119.8	C5_15—C1_15—C2_15	121 (3)
N1_5—C1_5—H1A_5	119.8	C5_15—C1_15—H1A_15	119.3
C1_5—C2_5—C3_5	117 (2)	C2_15—C1_15—H1A_15	119.3
C1_5—C2_5—H2_5	121.3	C1_15—C2_15—C3_15	119 (2)
C3_5—C2_5—H2_5	121.3	C1_15—C2_15—H2_15	120.3
C2_5—C3_5—C4_5	121 (2)	C3_15—C2_15—H2_15	120.3
C2_5—C3_5—H3_5	119.4	C4_15—C3_15—C2_15	114 (2)
C4_5—C3_5—H3_5	119.4	C4_15—C3_15—H3_15	123.1
C5_5—C4_5—C3_5	118 (2)	C2_15—C3_15—H3_15	123.1
C5_5—C4_5—H4_5	121.2	N1_15—C4_15—C3_15	121 (3)
C3_5—C4_5—H4_5	121.2	N1_15—C4_15—H4_15	119.6
N1_5—C5_5—C4_5	122 (2)	C3_15—C4_15—H4_15	119.6
N1_5—C5_5—H5_5	118.8	N1_15—C5_15—C1_15	121 (3)
C4_5—C5_5—H5_5	118.8	N1_15—C5_15—H5_15	119.4
C1_6—N1_6—C5_6	120.8 (19)	C1_15—C5_15—H5_15	119.4
C1_6—N1_6—H1_6	119.6		
			
O3_1—Mo1_1—O1_1—Mo2_1	−122.1 (15)	O1_3—Mo1_3—O6_3—S1_3	−15.6 (10)
O2_1—Mo1_1—O1_1—Mo2_1	132.1 (16)	O10_3—Mo1_3—O6_3—S1_3	−101.7 (10)
O6_1—Mo1_1—O1_1—Mo2_1	−33.2 (15)	Cl1_3—Mo1_3—O6_3—S1_3	177.2 (10)
O10_1—Mo1_1—O1_1—Mo2_1	45.6 (15)	O8_3—S1_3—O7_3—Mo2_3	50.3 (12)
Cl1_1—Mo1_1—O1_1—Mo2_1	7(2)	O9_3—S1_3—O7_3—Mo2_3	172.8 (9)
O4_1—Mo2_1—O1_1—Mo1_1	135.4 (16)	O6_3—S1_3—O7_3—Mo2_3	−71.4 (11)
O5_1—Mo2_1—O1_1—Mo1_1	−119.5 (15)	O5_3—Mo2_3—O7_3—S1_3	−67.8 (12)
O7_1—Mo2_1—O1_1—Mo1_1	46.7 (16)	O4_3—Mo2_3—O7_3—S1_3	125 (3)
O11_1—Mo2_1—O1_1—Mo1_1	−33.9 (15)	O1_3—Mo2_3—O7_3—S1_3	32.7 (11)
Cl2_1—Mo2_1—O1_1—Mo1_1	11 (2)	O11_3—Mo2_3—O7_3—S1_3	117.8 (12)
O9_1—S1_1—O6_1—Mo1_1	−81.6 (12)	Cl2_3—Mo2_3—O7_3—S1_3	−160.7 (11)
O8_1—S1_1—O6_1—Mo1_1	157.0 (9)	O12_3—S2_3—O10_3—Mo1_3	78.1 (17)
O7_1—S1_1—O6_1—Mo1_1	34.6 (14)	O13_3—S2_3—O10_3—Mo1_3	−157.1 (15)
O3_1—Mo1_1—O6_1—S1_1	76.3 (10)	O11_3—S2_3—O10_3—Mo1_3	−38.6 (16)
O2_1—Mo1_1—O6_1—S1_1	−131.6 (18)	O3_3—Mo1_3—O10_3—S2_3	−76.5 (12)
O1_1—Mo1_1—O6_1—S1_1	−24.4 (10)	O2_3—Mo1_3—O10_3—S2_3	135.2 (18)
O10_1—Mo1_1—O6_1—S1_1	−108.0 (10)	O1_3—Mo1_3—O10_3—S2_3	22.4 (12)
Cl1_1—Mo1_1—O6_1—S1_1	169.7 (10)	O6_3—Mo1_3—O10_3—S2_3	109.4 (12)
O9_1—S1_1—O7_1—Mo2_1	113.7 (17)	Cl1_3—Mo1_3—O10_3—S2_3	−170.2 (12)
O8_1—S1_1—O7_1—Mo2_1	−127.0 (16)	O12_3—S2_3—O11_3—Mo2_3	−102 (2)
O6_1—S1_1—O7_1—Mo2_1	−5(2)	O13_3—S2_3—O11_3—Mo2_3	137 (2)
O4_1—Mo2_1—O7_1—S1_1	−117.3 (18)	O10_3—S2_3—O11_3—Mo2_3	18 (3)
O5_1—Mo2_1—O7_1—S1_1	81 (3)	O5_3—Mo2_3—O11_3—S2_3	−99 (3)
O1_1—Mo2_1—O7_1—S1_1	−19.1 (17)	O4_3—Mo2_3—O11_3—S2_3	106 (2)
O11_1—Mo2_1—O7_1—S1_1	64.9 (17)	O1_3—Mo2_3—O11_3—S2_3	10 (2)
Cl2_1—Mo2_1—O7_1—S1_1	149.0 (18)	O7_3—Mo2_3—O11_3—S2_3	−75 (2)
O12_1—S2_1—O10_1—Mo1_1	146.7 (11)	Cl2_3—Mo2_3—O11_3—S2_3	−156 (2)
O13_1—S2_1—O10_1—Mo1_1	−92.0 (12)	C5_4—N1_4—C1_4—C2_4	2(3)
O11_1—S2_1—O10_1—Mo1_1	27.9 (14)	N1_4—C1_4—C2_4—C3_4	0(3)
O3_1—Mo1_1—O10_1—S2_1	68 (3)	C1_4—C2_4—C3_4—C4_4	0(3)
O2_1—Mo1_1—O10_1—S2_1	−138.0 (12)	C2_4—C3_4—C4_4—C5_4	−1(3)
O1_1—Mo1_1—O10_1—S2_1	−36.3 (11)	C1_4—N1_4—C5_4—C4_4	−3(3)
O6_1—Mo1_1—O10_1—S2_1	48.0 (11)	C3_4—C4_4—C5_4—N1_4	2(3)
Cl1_1—Mo1_1—O10_1—S2_1	129.8 (11)	C5_5—N1_5—C1_5—C2_5	−3(3)
O12_1—S2_1—O11_1—Mo2_1	−120.1 (16)	N1_5—C1_5—C2_5—C3_5	3(3)
O13_1—S2_1—O11_1—Mo2_1	118.3 (15)	C1_5—C2_5—C3_5—C4_5	−4(3)
O10_1—S2_1—O11_1—Mo2_1	−1.8 (19)	C2_5—C3_5—C4_5—C5_5	3(3)
O4_1—Mo2_1—O11_1—S2_1	−98 (3)	C1_5—N1_5—C5_5—C4_5	3(3)
O5_1—Mo2_1—O11_1—S2_1	97.8 (17)	C3_5—C4_5—C5_5—N1_5	−3(3)
O1_1—Mo2_1—O11_1—S2_1	−2.7 (16)	C5_6—N1_6—C1_6—C2_6	−5(3)
O7_1—Mo2_1—O11_1—S2_1	−86.0 (17)	N1_6—C1_6—C2_6—C3_6	2(3)
Cl2_1—Mo2_1—O11_1—S2_1	−168.1 (17)	C1_6—C2_6—C3_6—C4_6	0(3)
O2_2—Mo1_2—O1_2—Mo2_2	−126.3 (16)	C2_6—C3_6—C4_6—C5_6	1(3)
O3_2—Mo1_2—O1_2—Mo2_2	126.4 (16)	C1_6—N1_6—C5_6—C4_6	5(3)
O10_2—Mo1_2—O1_2—Mo2_2	37.0 (16)	C3_6—C4_6—C5_6—N1_6	−3(3)
O6_2—Mo1_2—O1_2—Mo2_2	−41.3 (16)	C5_7—N1_7—C1_7—C2_7	−4(3)
Cl1_2—Mo1_2—O1_2—Mo2_2	0(2)	N1_7—C1_7—C2_7—C3_7	0(4)
O5_2—Mo2_2—O1_2—Mo1_2	−132.0 (16)	C1_7—C2_7—C3_7—C4_7	1(3)
O4_2—Mo2_2—O1_2—Mo1_2	122.9 (16)	C2_7—C3_7—C4_7—C5_7	1(3)
O7_2—Mo2_2—O1_2—Mo1_2	32.4 (16)	C1_7—N1_7—C5_7—C4_7	6(3)
O11_2—Mo2_2—O1_2—Mo1_2	−45.9 (16)	C3_7—C4_7—C5_7—N1_7	−4(3)
Cl2_2—Mo2_2—O1_2—Mo1_2	−5(3)	C5_8—N1_8—C1_8—C2_8	4(4)
O8_2—S1_2—O6_2—Mo1_2	−104.8 (16)	N1_8—C1_8—C2_8—C3_8	−1(3)
O9_2—S1_2—O6_2—Mo1_2	132.5 (14)	C1_8—C2_8—C3_8—C4_8	0(3)
O7_2—S1_2—O6_2—Mo1_2	15.7 (17)	C2_8—C3_8—C4_8—C5_8	−2(4)
O2_2—Mo1_2—O6_2—S1_2	109.7 (15)	C1_8—N1_8—C5_8—C4_8	−6(4)
O3_2—Mo1_2—O6_2—S1_2	−107 (3)	C3_8—C4_8—C5_8—N1_8	4(3)
O1_2—Mo1_2—O6_2—S1_2	7.6 (14)	C5_9—N1_9—C1_9—C2_9	5(5)
O10_2—Mo1_2—O6_2—S1_2	−76.8 (15)	N1_9—C1_9—C2_9—C3_9	−5(6)
Cl1_2—Mo1_2—O6_2—S1_2	−157.9 (15)	C1_9—C2_9—C3_9—C4_9	10 (6)
O8_2—S1_2—O7_2—Mo2_2	84.9 (15)	C2_9—C3_9—C4_9—C5_9	−14 (5)
O9_2—S1_2—O7_2—Mo2_2	−152.6 (11)	C1_9—N1_9—C5_9—C4_9	−11 (4)
O6_2—S1_2—O7_2—Mo2_2	−34.9 (15)	C3_9—C4_9—C5_9—N1_9	15 (4)
O5_2—Mo2_2—O7_2—S1_2	125.1 (18)	C5_10—N1_10—C1_10—C2_10	2(3)
O4_2—Mo2_2—O7_2—S1_2	−78.3 (12)	N1_10—C1_10—C2_10—C3_10	2(3)
O1_2—Mo2_2—O7_2—S1_2	20.9 (12)	C1_10—C2_10—C3_10—C4_10	−5(3)
O11_2—Mo2_2—O7_2—S1_2	104.0 (12)	C2_10—C3_10—C4_10—C5_10	3(3)
Cl2_2—Mo2_2—O7_2—S1_2	−171.9 (12)	C3_10—C4_10—C5_10—N1_10	1(3)
O13_2—S2_2—O10_2—Mo1_2	−122.3 (16)	C1_10—N1_10—C5_10—C4_10	−3(3)
O11_2—S2_2—O10_2—Mo1_2	−1(2)	C5_11—N1_11—C1_11—C2_11	−3(3)
O12_2—S2_2—O10_2—Mo1_2	118.9 (17)	N1_11—C1_11—C2_11—C3_11	0(3)
O2_2—Mo1_2—O10_2—S2_2	110 (2)	C1_11—C2_11—C3_11—C4_11	3(3)
O3_2—Mo1_2—O10_2—S2_2	−99.5 (17)	C2_11—C3_11—C4_11—C5_11	−2(3)
O1_2—Mo1_2—O10_2—S2_2	1.7 (17)	C1_11—N1_11—C5_11—C4_11	3(3)
O6_2—Mo1_2—O10_2—S2_2	87.0 (17)	C3_11—C4_11—C5_11—N1_11	0(3)
Cl1_2—Mo1_2—O10_2—S2_2	168.3 (18)	C5_12—N1_12—C1_12—C2_12	3(5)
O13_2—S2_2—O11_2—Mo2_2	99.1 (14)	N1_12—C1_12—C2_12—C3_12	−5(5)
O12_2—S2_2—O11_2—Mo2_2	−139.1 (13)	C1_12—C2_12—C3_12—C4_12	−1(5)
O10_2—S2_2—O11_2—Mo2_2	−19.0 (17)	C2_12—C3_12—C4_12—C5_12	8(4)
O5_2—Mo2_2—O11_2—S2_2	129.1 (14)	C3_12—C4_12—C5_12—N1_12	−11 (4)
O4_2—Mo2_2—O11_2—S2_2	−69 (3)	C1_12—N1_12—C5_12—C4_12	6(5)
O1_2—Mo2_2—O11_2—S2_2	28.6 (13)	C5_13—N1_13—C1_13—C2_13	−1(3)
O7_2—Mo2_2—O11_2—S2_2	−56.5 (13)	N1_13—C1_13—C2_13—C3_13	−1(3)
Cl2_2—Mo2_2—O11_2—S2_2	−137.7 (13)	C1_13—C2_13—C3_13—C4_13	2(3)
O5_3—Mo2_3—O1_3—Mo1_3	125.4 (14)	C2_13—C3_13—C4_13—C5_13	−2(3)
O4_3—Mo2_3—O1_3—Mo1_3	−130.8 (16)	C1_13—N1_13—C5_13—C4_13	1(3)
O11_3—Mo2_3—O1_3—Mo1_3	−41.3 (15)	C3_13—C4_13—C5_13—N1_13	1(3)
O7_3—Mo2_3—O1_3—Mo1_3	37.7 (14)	C5_14—N1_14—C1_14—C2_14	0(4)
Cl2_3—Mo2_3—O1_3—Mo1_3	−1(2)	N1_14—C1_14—C2_14—C3_14	−2(4)
O3_3—Mo1_3—O1_3—Mo2_3	123.4 (15)	C1_14—C2_14—C3_14—C4_14	−2(4)
O2_3—Mo1_3—O1_3—Mo2_3	−132.1 (14)	C2_14—C3_14—C4_14—C5_14	7(6)
O10_3—Mo1_3—O1_3—Mo2_3	31.5 (14)	C3_14—C4_14—C5_14—N1_14	−8(6)
O6_3—Mo1_3—O1_3—Mo2_3	−43.5 (14)	C1_14—N1_14—C5_14—C4_14	5(5)
Cl1_3—Mo1_3—O1_3—Mo2_3	−6(2)	C5_15—C1_15—C2_15—C3_15	−1(4)
O8_3—S1_3—O6_3—Mo1_3	−63.0 (12)	C1_15—C2_15—C3_15—C4_15	−1(3)
O9_3—S1_3—O6_3—Mo1_3	173.8 (9)	C5_15—N1_15—C4_15—C3_15	−6(4)
O7_3—S1_3—O6_3—Mo1_3	57.9 (11)	C2_15—C3_15—C4_15—N1_15	4(3)
O3_3—Mo1_3—O6_3—S1_3	−127 (2)	C4_15—N1_15—C5_15—C1_15	4(4)
O2_3—Mo1_3—O6_3—S1_3	85.9 (11)	C2_15—C1_15—C5_15—N1_15	−1(4)

### Hydrogen-bond geometry (Å, °)

**Table:**

*D*—H···*A*	*D*—H	H···*A*	*D*···*A*	*D*—H···*A*
N1_4—H1_4···O8_2	0.88	2.18	3.01 (3)	157
N1_5—H1_5···O9_1	0.88	2.43	3.15 (2)	139
N1_6—H1_6···O12_2	0.88	2.50	3.32 (3)	157
N1_7—H1_7···O6_2^i^	0.88	2.11	2.99 (2)	176
N1_8—H1_8···O6_3	0.88	2.13	2.90 (2)	146
N1_8—H1_8···O10_3	0.88	2.39	3.09 (2)	137
N1_9—H1_9···O7_2	0.88	2.03	2.86 (3)	157
N1_9—H1_9···O9_2	0.88	2.41	2.94 (2)	119
N1_10—H1_10···O4_3	0.88	2.34	3.01 (3)	133
N1_11—H1_11···O13_1	0.88	2.04	2.84 (2)	149
N1_11—H1_11···O11_1	0.88	2.64	3.18 (2)	121
N1_12—H1_12···O5_3	0.88	2.00	2.74 (3)	141
N1_12—H1_12···O8_3	0.88	2.45	3.07 (3)	128
N1_13—H1_13···O13_1	0.88	1.85	2.722 (18)	173
N1_14—H1_14···O8_3	0.88	2.25	3.09 (3)	160
N1_15—H1_15···O9_2^ii^	0.88	2.25	3.02 (2)	146

Symmetry codes: (i) *x*, *y*, *z*−1; (ii) *x*+1/2, −*y*+1/2, *z*−1/2.

### Table 2 Geometrical parameters (Å,°) for the three crystallographically independent molybdenum clusters

**Table:**

Mo—O1	1.857 (13)–1.969 (14)
Mo—O_terminal_	1.678 (11)–1.717 (12)
Mo—O_sulfato_	2.164 (16)–2.271 (14)
Mo—Cl	2.271 (14)–2.442 (5)
O_terminal_—Mo—O_terminal_	102.2 (7)–104.3 (8)
*cis*-O_terminal_—Mo—O_sulfato_	86.4 (6)–92.6 (7)
*trans*-O_terminal_—Mo—O_sulfato_	162.5 (6)–169.4 (4)
O_terminal_—Mo—O1	96.4 (7)–101.6 (5)
O_terminal_—Mo—Cl	91.2 (5)–97.4 (6)
O_sulfato_—Mo—O_sulfato_	74.8 (5)–79.8 (6)
O_sulfato_—Mo—O1	82 (5)–85.9 (5)
O_sulfato_—Mo—Cl	77.8 (3)–82.6 (3)
O1—Mo—Cl	158 (4)–159.4 (3)
Mo—O1—Mo	149.3 (7)–155 (6)

Notes: O1 stands for the µ-O oxygen atom; O_terminal_ corresponds to O2, O3, O4 and O5; O_sulfato_ corresponds to O6, O7, O10 and O11.
